# An SPR Sensor Chip Based on Peptide-Modified Single-Walled Carbon Nanotubes with Enhanced Sensitivity and Selectivity in the Detection of 2,4,6-Trinitrotoluene Explosives

**DOI:** 10.3390/s18124461

**Published:** 2018-12-17

**Authors:** Jin Wang, Sanyang Du, Takeshi Onodera, Rui Yatabe, Masayoshi Tanaka, Mina Okochi, Kiyoshi Toko

**Affiliations:** 1 Research and Development Center for Five-Sense Devices, Kyushu University, 744 Motooka, Nishiku, Fukuoka 819-0395, Japan; toko@ed.kyushu-u.ac.jp; 2 JST, ImPACT, Sanban-cho 5, Chiyoda-ku, Tokyo 102-0075, Japan; du.sanyang.748@s.kyushu-u.ac.jp (S.D.); onodera@ed.kyushu-u.ac.jp (T.O.); yatabe.rui.678@m.kyushu-u.ac.jp (R.Y.); m_tanaka@chemeng.titech.ac.jp (M.T.); okochi@chemeng.titech.ac.jp (M.O.); 3 Graduate School of Information Science and Electrical Engineering, Kyushu University, 744 Motooka, Nishiku, Fukuoka 819-0395, Japan; 4 Department of Chemical Science and Engineering, Tokyo Institute of Technology, 2-12-1, O-okayama, Meguro-ku, Tokyo 152-8552, Japan; 5 Institute for Advanced Study, Kyushu University, 744 Motooka, Nishiku, Fukuoka 819-0395, Japan

**Keywords:** SPR, TNTHCDR3, SWCNTs, π-stacking, TNT analogues

## Abstract

In this study, we developed a surface plasmon resonance (SPR) sensor chip based on 2,4,6-trinitrotoluene (TNT) recognition peptide-modified single-walled carbon nanotubes (SWCNTs). The carboxylic acid-functionalized SWCNTs were immobilized on a 3-aminopropyltriethoxysilane (APTES)-modified SPR Au chip surface. Through π-stacking between the aromatic amino acids and SWCNTs, the TNT recognition peptide TNTHCDR3 was immobilized onto the surface of the SWCNTs. The peptide–SWCNTs-modified sensor surface was confirmed and evaluated by atomic force microscope (AFM) observation. The peptide–SWCNTs hybrid SPR sensor chip exhibited enhanced sensitivity with a limit of detection (LOD) of 772 ppb and highly selective detection compared with commercialized carboxymethylated dextran matrix sensor chips.

## 1. Introduction

Surface plasmon resonance (SPR) is a powerful technique for the study of large biomolecule interactions in real time with a simple and rapid analysis procedure. SPR-based biosensing is widely used in medical diagnostics, pharmaceutical research, and food research, measuring the adsorption and desorption coefficients of the biomolecules. The principle of SPR is based on the fact that the signal is proportional to the refractive index (RI) of the medium near gold metal films. Therefore, any change in the environment of the medium 300 nm out from the surface will significantly change the signal [1,2,3]. Great effects have been achieved in enhancing SPR signals using gold nanoparticles/rods, quantum dots, graphene oxide, or carbon nanotubes [1,4,5,6,7,8,9]. Self-assembled monolayers and dextran polymers are often used for immobilizing the ligand molecules on the gold surface. However, these extensive effects on the enhancement of the SPR signal were still mainly used for the direct detection of large molecules, such as proteins, DNA, and antibodies, and for the indirect detection (competitive detection) of small molecules [6]. The detection of low concentrations of small molecules via direct detection is still challenging because of their insufficient mass for measurable change to the RI.

The synthesis and screening of 2,4,6-trinitrotoluene (TNT)-binding peptides for the direct detection of TNT explosives using SPR sensors has been previously investigated [10,11,12]. However, obtaining high sensitivity and selectivity has been hampered by SPR sensors without signal amplification. Therefore, we aim to develop a peptide-functionalized single-walled carbon nanotubes (SWCNTs) hybrid-anchored SPR sensor chip for enhanced sensitivity and selectivity. SWCNTs consist of only one graphene layer rolled in on itself, forming a tube shape; exhibit unique electrical/optical properties, and excellent biocompatibility; and are recognized as an excellent candidate for SPR enhancement [13,14,15,16]. Moreover, SWCNTs can be easily functionalized for bioreceptors, such as DNA strands or peptides, through π-stacking [17,18]. SWCNTs with diameters of a few nanometers keep the interaction between the bioreceptor and the analytes in the effective range where the changes in the refractive index within the electric field could be measured sensitively. In this study, TNTHCDR3 (15-amino acid sequence: ARGYSSFIYWFFDFC), demonstrated to be a TNT recognition peptide [10,11,12], was enriched in three kinds of aromatic amino acids (Y, tyrosine; W, tryptophan; and F, phenylalanine), which bind to the SWCNTs surface through noncovalent bonding via π–π interactions. The results reveal that the proposed method offers enhanced sensitivity, selectivity, and long-term stability in the detection of TNT explosives.

## 2. Materials and Methods

### 2.1. Materials and Reagents

TNT and RDX (Research Development eXplosive)solutions (10.03 mg/mL) dissolved in pure DMF (*N,N*-dimethylformamide) were obtained from Chugoku Kayaku, Co., Ltd., Kure, Japan and were freshly diluted with phosphate-buffered saline (PBS, 0.1 M, pH 7.4) containing 0.05% Tween 20 (T) and 1% DMF, as required. 2,4-dinitrophenyl glycine, 2,6-dinitrotoluene, and 4-nitrobenzoyl-glycyl-glycine were purchased from Tokyo Chemical Industry, Tokyo, Japan. 3-aminopropyltriethoxysilane (APTES) was purchased from Sigma-Aldrich, St. Louis, MO, USA. The SPR sensor chip (bare gold) and sensor chip CM7 (carboxymethylated dextran, designed for the detection of small molecules) were purchased from GE Healthcare, Uppsala, Sweden. Sodium hydroxide solution (NaOH) and potassium hydroxide solution (KOH) were purchased from FUJIFILM Wako Pure Chemical Industries, Tokyo, Japan. *N,N′*-dicyclohexylcarbodiimide (DCC) was purchased from Sigma-Aldrich, St. Louis, MO, USA. Single-walled carbon nanotubes with carboxylic acid functionalization (SWCNTs) were purchased from Sigma-Aldrich, St. Louis, MO, USA. Piranha solution consisting of sulfuric acid (H_2_SO_4_) and hydrogen peroxide (H_2_O_2_) solution was purchased from Sigma-Aldrich, St. Louis, MO, USA (piranha solution cleaning must be operated in a fume hood with adequate safety protection). Potassium ferricyanide (K_3_[Fe(CN)_6_]) and potassium chloride (KCl), used for the cyclic voltammetry (CV) of the SWCNTs, were purchased from FUJIFILM Wako Pure Chemical Industries, Tokyo, Japan. All the other chemicals were purchased either from Tokyo Chemical Industry, Tokyo, Japan, or FUJIFILM Wako Pure Chemical Industries, Tokyo, Japan. All the aqueous solutions were prepared using Milli-Q deionized water (18 MΩ) from a Milli-Q system (Millipore Corporation, Billerica, MA, USA).

### 2.2. Fabrication Procedure of the Peptide-Functionalized SPR Sensor Chip Surface

**Figure 1:**
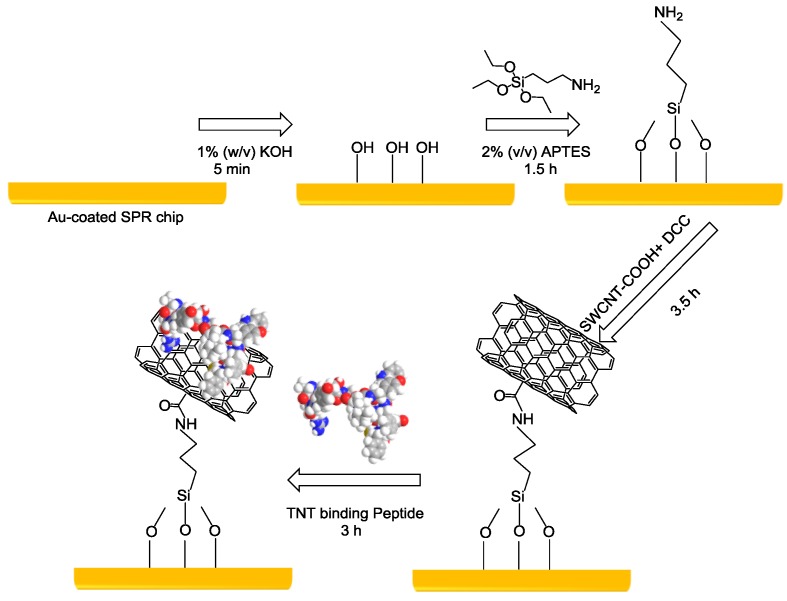
A schematic illustration of the immobilization procedure of the 2,4,6-trinitrotoluene (TNT)-binding peptide TNTHCDR3 on the surface of a single-walled carbon nanotube (SWCNT)-based surface plasmon resonance (SPR) gold-coated chip.

The TNT-binding peptide was determined to be TNTHCDR3 (ARGYSSFIYWFFDFC) according to our previous work [10,11,12]. A SPR sensor chip covered with a 50-nm gold layer was used for multistep functionalization (Figure 1). The surface modification procedure was conducted as follows. First, the bare gold SPR sensor chip was cleaned with a mixed solution consisting of ammonia solution, hydrogen peroxide, and Milli-Q water with a volume ratio of 2:2:10 at 90 °C for 20 min. Next, the piranha solution was used to remove most of the contaminates on the gold surface to render it super clean. The gold chip was immersed in a 1% w/v KOH solution (90 µL) for at least 5 min to generate the hydroxyl groups. A 2% (*v*/*v*) APTES solution (100 µL) was added onto the surface for 1.5 h to generate the amine groups. For preparing the SWCNT supernatant, 0.2 mg of carboxylic acid-functionalized SWCNTs were dispersed in 5 mL DMF with 0.5 mg of DCC to convert the carboxylic groups into active carbodiimide esters [19,20]. The 5-mL DMF solution with 0.2 mg of carboxylic acid-functionalized SWCNTs was firstly sonicated for 20 min in an ultrasonic bath (chilled ice was added to control the bath temperature at 25 °C). Then, the suspension was carefully extracted for centrifugation for 40 min at 13,200 rpm. A stable SWCNT supernatant with no precipitate was obtained and was ready for use for the sensor chip surface fabrication. Then, the APTES-modified sensor surface was immersed into the SWCNTs with the active carbodiimide ester solution forming amide bonds for 3.5 h. Subsequently, TNTHCDR3 peptide at a concentration of 600 ppm (prepared in 90% DMF solvent) was non-covalently immobilized on the SWCNT surfaces through π–π interaction. The evaluation of the peptide–SWCNT-based SPR sensor chip was performed using a Biacore X100 instrument. Then, 8 ascending TNT sample solutions (i.e., 0.8 ppm, 1.6 ppm, 3.2 ppm, 6.3 ppm, 12.5 ppm, 25 ppm, 50 ppm, and 100 ppm) were prepared and injected in triplicate for 80 s at a flow rate of 10 μL/min. Mild regeneration of the sensor surface was performed using 5 mM NaOH at the end of each measurement. A 1% DMF solution was added into the running buffer solution to eliminate the bulk effects caused by the DMF. Flow and injection of the running buffer solution were conducted for system priming before each sample measurement. DMSO solution (5% and 10%) was applied to super clean the system after the total measurements.

## 3. Results and Discussion

### 3.1. TNT Recognition Peptide (TNTHCDR3) and SWCNT-Modified SPR Sensor Chip

**Figure 2:**
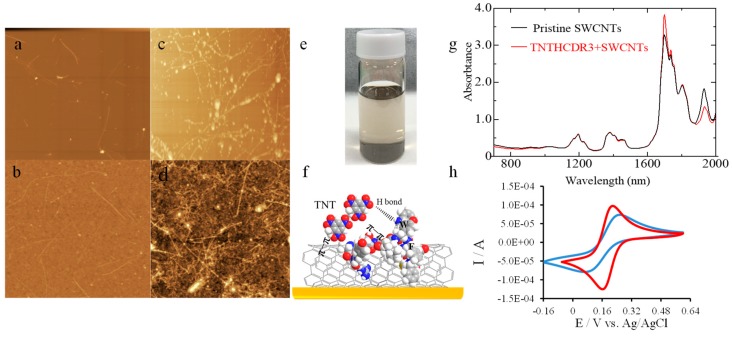
Atomic force microscope (AFM) analysis of the peptide–SWCNT-based SPR sensor surface fabrication procedure (5 μm × 5 μm) (a~d). (**a**) Pristine SWCNTs on a mica substrate; (**b**) SWCNTs on a 3-aminopropyltriethoxysilane (APTES)-based SPR Au-coated chip; (**c**) TNTHCDR3 anchored on SWCNTs on a mica substrate; (**d**) TNTHCDR3 anchored on a SWCNT-based gold surface; (**e**) Image of the mixed solution of TNTHCDR3 and SWCNTs; (**f**) Illustration of the π–π interaction between TNTHCDR3 and SWCNTs, as well as the mechanism of 2,4,6-trinitrotoluene (TNT) binding to the peptide–SWCNT hybrids; (**g**) FT-NIR absorbance spectra of pristine SWCNTs and TNTHCDR3 peptide-functionalized SWCNTs; and (**h**) Cyclic voltammetry (CV) curves of bare gold (blue) and the SWCNT-modified gold chip (red).

Three kinds of aromatic amino acids (Y, tyrosine; W, tryptophan; and F, phenylalanine) in the TNT recognition peptide sequence TNTHCDR3 (ARGYSSFIYWFFDFC) take an active part in binding to the TNT molecules via hydrogen bonding (W) and π electron-mediated effects (Y, W, and F) [21,22]. Using noncovalent bonding via π–π interactions without damaging the intrinsic properties of the SWCNTs, the TNT recognition peptide was anchored onto the SWCNT-based SPR sensor chip surface. The surface structure and morphology of the peptide–SWCNT hybrid were analyzed by AFM. The morphology of the pristine SWCNTs (40 µg/mL in DMF) on a mica substrate and on a SPR gold-coated chip was determined by AFM and is shown in Figure 2a,b. Well-dispersed SWCNTs could be observed on both surfaces. Noncovalent binding via π–π interaction between TNTHCDR3 and SWCNTs is demonstrated in Figure 2c (on a mica substrate) and Figure 2d (on a SPR gold surface). In those figures, the bright dot, along with the SWCNTs, indicates the peptide unit. The AFM analysis results demonstrated the conformation of the hybrid material on the surface. The immobilization procedure of the SWCNTs on the APTES-based gold surface was described above. A homogeneous stable suspension formed by π–π interactions between the aromatic amino acids in the TNTHCDR3 peptide and the SWCNTs can be observed in Figure 2e. Figure 2f simulates the interaction between the TNTHCDR3 peptide and the SWCNTs, in agreement with the mechanism reported in a previous study [23]. Meanwhile, the comparison of the FT-NIR absorbance spectra in Figure 2g between the pristine SWCNTs and the TNTHCDR3 peptide-functionalized SWCNTs in DMF solvent clearly shows that good dispersion occurred without aggregation [24]. To prove that the SWCNTs were successfully immobilized on the APTES-based SPR gold surface shown in Figure 2b, CV was performed for comparison with and without a SWCNT-modified gold surface. The scan rate was set to 100 m Vs^-1^. K_3_[Fe(CN)_6_] (1 mM), and KCl (50 mM) in 10-mM phosphate buffer (pH 7.4) was used as the electrolyte [19]. Clearly, the SWCNT-modified gold surface offered greater ferricyanide reduction peak currents compared with the surface without SWCNT modification. The CV results reflect the larger surface area of the SWCNT-modified gold surface and the enhancement of the electron transfer (Figure 2h).

### 3.2. Performance of the Peptide–SWCNT-Based SPR Sensor

**Figure 3:**
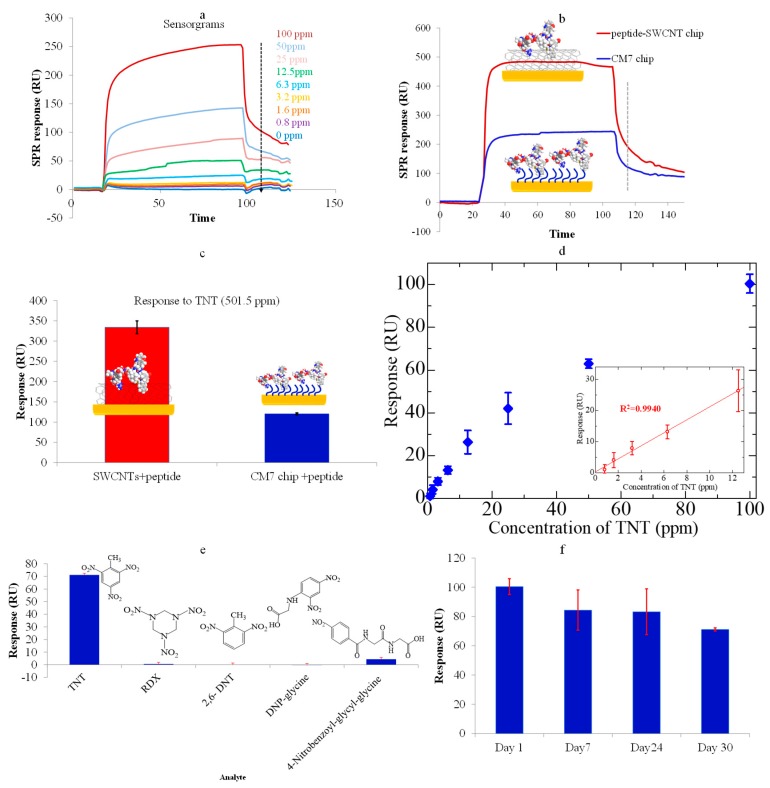
(**a**) The real-time sensorgrams of the peptide–SWCNT hybrids corresponding to TNT concentrations; (**b**) The real-time sensorgrams of two kinds of sensor chips corresponding to TNT explosives at a concentration of 501.5 ppm; (**c**) A comparison of the responses of two kinds of sensor chips to TNT solutions; (**d**) The responses of the SWCNT–peptide chip and CM7 chip to TNT (Inset: A plot of the response corresponding to various TNT concentrations in the highly linear range). The error bar indicates the calculated standard deviation (*n* = 3); (**e**) The response of the TNTHCDR3-anchored SWCNT sensor chip to 100 ppm solutions of TNT, RDX, 2,6-DNT, 4-nitrobenzoyl-glycyl-glycine, and DNP-glycine. The error bar indicates the calculated standard deviation (*n* = 3); (**f**) The stability of the sensor chip over a duration of 1 month (response to 100 ppm TNT solution).

Figure 3a shows the real-time sensorgrams of the peptide–SWCNT-based SPR to various TNT concentrations (0 ppm~100 ppm). Compared with the results of using a conventional dextran chip (CM7 chip) in Figure 3b,c, the present peptide–SWCNT hybrid sensor chip exhibited a significantly higher response, revealing that SWCNTs are promising materials for low-molecular-weight (LMW) signal enhancement in lower concentrations [11,12]. Figure 3d shows the relationship between the SPR resonance unit (RU) and various concentrations of TNT explosives. The results in the Figure 3d inset demonstrate a high linearity in the concentration range 0.8~12.5 ppm (R^2^ = 0.9940). The limit of detection (LOD) calculated was 772 ppb (3.4 µM) in the background solution (3σ); this LOD is competitive against using the peptide as a TNT receptor-based EIS (electrochemical impedance spectroscopy) (1 µM), SERS (surface enhanced raman scattering) (100 pM), or QCM (quartz crystal microbalance) biosensor (not mentioned) [25,26,27]. However, the results revealed that the SPR sensor was still less sensitive when detecting small molecules with direct measurements, because they do not have sufficient mass to produce sufficiently high measurable signals at very low concentrations, as compared with using electric sensing methods, such as nanowire/SWCNTs-based FET (field effect transistor) with ppq level [28], nM level [29], or fM level [30] or three-dimensional (3D) microcantilevers with ppt level [31], using organic or bioreceptors for ultra-highly sensitive and selective TNT detection. 

Highly specific TNT detection and long-term stability were obtained by using the peptide–SWCNT-based SPR biosensor. The results in Figure 3e clearly show that the TNT explosive analogues, including DNP-glycine, 2,6-DNT, RDX, and 4-nitrobenzoyl-glycyl-glycine (at 100 ppm concentration), had very small, negligible responses compared with the significant response to the TNT. The stability of the peptide–SWCNT hybrid-based SPR sensor was also evaluated over a period of 1 month. The concentration of the TNT sample was chosen to be 100 ppm, which required mild regeneration to the baseline (16 regeneration cycles using 5-mM NaOH solution for 10 s each time). The sensor response gradually decreased because of the reduced peptide activity mainly caused by the surface regeneration process or other effects during the storage period. As shown in Figure 2f, the sensor response to 100 ppm TNT explosives was about 84.0% of its original value after 1 week, 83.0% after 3 weeks, and 70.8% after 4 weeks. The results reveled an acceptable binding ability of the peptide-based SWCNT hybrid sensor chip. 

## 4. Conclusions

To improve the performance of the SPR sensor for LMW detection, we presented a SPR sensor chip based on a peptide-modified SWCNT hybrid with long-term stability for enhanced sensitivity and high selectivity in the detection of TNT explosives. The sensor response was significantly amplified by the peptide–SWCNT hybrid, and nonspecific binding on the surface by other analogues was drastically reduced compared with that for the commercialized Biacore SPR CM7 sensor chip, as shown through direct determinations based on a previous investigation [11]. However, for ultra-highly sensitive detection of small molecule compounds, a peptide–SWCNT hybrid-based chemiresistor or FET platform is expected to be developed in future work based on the present progress.
